# Digital Phenotyping and Dynamic Monitoring of Adolescents Treated for Cancer to Guide Intervention: Embracing a New Era

**DOI:** 10.3389/fonc.2021.673581

**Published:** 2021-06-28

**Authors:** Johanna M. C. Blom, Chiara Colliva, Cristina Benatti, Fabio Tascedda, Luca Pani

**Affiliations:** ^1^ Department of Biomedical, Metabolic and Neural Sciences, University of Modena and Reggio Emilia, Modena, Italy; ^2^ Center for Neuroscience and Neurotechnology, University of Modena and Reggio Emilia, Modena, Italy; ^3^ Azienda Unità Sanitaria Locale di Modena, Distretto di Carpi, Carpi, Italy; ^4^ Department of Life Sciences, University of Modena and Reggio Emilia, Modena, Italy; ^5^ Department of Psychiatry and Behavioral Sciences, University of Miami, Miami, FL, United States; ^6^ VeraSci., Durham, NC, United States

**Keywords:** behavioral toxicity, follow-up, dynamic monitoring, digital phenotype, adolescent cancer, network analysis

## Introduction

Adolescents diagnosed with and treated for cancer represent a particular vulnerable patient population with unique and complex medical and psychosocial needs that extend beyond the completion of treatment (1–4). These cancer patients are in the most critical phases of their development (5–7), with brain, body, mind, and social environment in constant transformation conferring vulnerability to some individuals and resilience to others. Adolescence is characterized by a heightened incidence (and first onset) of various mental illnesses (8–10). Cancer during adolescence influences peer relationships, changes mood and behavior, interferes with cognitive functioning, educational achievement, and increases the risk for the onset of psychopathology (11, 12). Furthermore, treatment is often accompanied by numerous physical and psychological symptoms such as nausea, vomiting, sleep disturbance, fatigue, pain, changes in body weight and body-image (2), treatment-induced psychosis, depression, and anxiety. Importantly, peer interactions in young cancer patients is often suddenly interrupted putting them at a higher risk for the development of emotional and behavioral problems. Even though many of these concerns remain largely unattended because of a lack of timely detection and management (13, 14), recent data indicate a growing awareness of the specific needs of this patient population suggesting that a concerted effort should be made to develop age-appropriate resources that could help them manage their illness and its treatment (15).

When asked, adolescents indicate five domains of concern: 1) physical, emotional, behavioral, and mood problems related to treatment; 2) psychosocial issues; 3) present and future adjustments once the therapy has finished; 4) transition, organization, and management of survivorship; and 5) need to be better connected, involved, and informed (16–20).

Given this, it is vital to capture, as early as possible, the biological, psychological, emotional, behavioral, and social modifications in young cancer patients to expand our day-to-day understanding of the factors affecting their vulnerability.

Most importantly, reviews and guidelines based on best practice stress the fact that cancer in adolescents does not always have chronic negative outcome especially if we understand which individuals are at heightened risk (20). Any issue in the five domains of concern listed above must be detected early to put timely and preventive measures into place (21). Adolescent cancer patients represent an enormously heterogenous group and while most somatic treatments are accompanied by the development of acute or chronic unwanted effects (2, 4), we currently lack the capacity to distinguish individual trajectories leading to increased vulnerability or resilience. A paradigm shift is essential to detect the distinct clinical pathways of high-risk individuals (22–24).

Current innovative digital approaches to patient care and monitoring offer a unique opportunity to create predictive models of individual vulnerability based on the integration and interdependencies of diverse sources of information. The analysis of the data contained in these platforms allow the projection of potential outcomes and disease trajectories, identifying those patients that are progressing from generic vulnerability to becoming at high risk for a negative event.

Digital tools may have numerous potential benefits compared to traditional assessments: they are non-invasive, ecological, do not demand extra efforts, and provide continuous access which offers timely understandings of the emotional, behavioral, cognitive, and treatment related changes. Also, while not all studies underline a clear benefit, some of them indicate that they are unrestricted by time and place, and offer immediate access to data and intermediate endpoints, reduce stressful visits, remove barriers to access to care (fear, isolation), stimulate patient empowerment, and, most importantly, help in the identification of high-risk patients and their risk stratification (23–26). As a result, the integration of various digital tools in a “toolbox,” would offer a concrete opportunity to modify, replace, or accompany the current more categorical approach and shift to a multimodal dimensional methodology (27, 28) in which evidence is gathered from different domains, ranging from subtle neurocognitive disfunction (29–33) to biomarkers. Ultimately, the multidimensional continuous recording of data and especially their dynamic relationships would become an integral part of the care plan and serve as specifiers of an high-risk status (34, 35).

## A Digital Approach in Understanding Acute and Chronic Behavioral Toxicity in Adolescents Treated for Cancer

Progress in the use of digital technologies and data analytics have created unmatched prospects to evaluate and alter health behavior and outcomes. Young adults and adolescents display a high compliance with the digital world surrounding them and are very comfortable with modern technologies (36, 37). However, while acceptance of digital tools is high (19), many health applications and tools are not specific for adolescents. Apps for various pathological conditions exist, supporting the life of patients suffering from diabetes, obesity, hypertension, and psychopathology (38). Several apps, mostly focused on symptom tracking and monitoring quality of life, exist for adult cancer patients while very few have been developed with the adolescent patient in mind (25, 39–44).

## Digital Phenotyping as Part of a Digital Toolbox

Adolescents with cancer consider psychological, emotional, cognitive, and social problems, issues of major concern (11–13). Touching the right cords is fundamental if we want to protect and help them. Digital phenotyping offers promising features for use in this patient group because it may allow objective and continuous measurements, documenting, and quantifying mood, energy level, and cognition in a non-invasive manner using personal digital devices (such as smartphones or wearables). This ecological approach centered on patients’ everyday lives has proven valuable when assessing symptomatology. Analysis by real-time algorithms allows for checking and detecting alarming pattern changes in daily functioning and predict when an individual shifts from being at risk to a patient that is about to experience an episode in need of immediate care (32, 33). Thus, digital phenotyping provides a period of long continuous surveillance of disease related moderators and mediators elucidating the temporal dynamics between specific biological mechanisms and changes in mood and behaviors.

Digital phenotyping, however, should be taken as just one of the instruments of a digital health toolbox. If we want a comprehensive approach, digital phenotyping must be accompanied by other participatory and communication tools, which include various objective biological markers that can be detected by sensors, wearables, and devices, such as smartwatches, rings, and fitness trackers (38, 45–47). Digital biomarkers and dynamic monitoring of functions, such as heartbeat, weight, blood pressure, temperature, sleep patterns, fatigue, and pain, provide important additional parameters related to disease, treatment related side effects, and effectiveness of treatment.

Lastly, the toolbox should also include the use of subjective measures, such as patient reported outcomes since they capture different nonobjective aspects of the same clinical construct, for instance fatigue (22, 25, 44). This information requires the active participation of the individual including symptom reporting or the use of activity diaries tracking personal impressions and perceptions, or responding to periodic questionnaires related to personal wellbeing and psychosocial health. A final advantage of an integrated digital approach is putting the adolescents at the very center of the data generation to fulfill their much-felt need to be informed, educated, and connected (20). In addition, providing access to information, videos, tutorials offering access to support groups or patient advocacy organizations (if desired) will connect and empower them even more.

**Figure 1:**
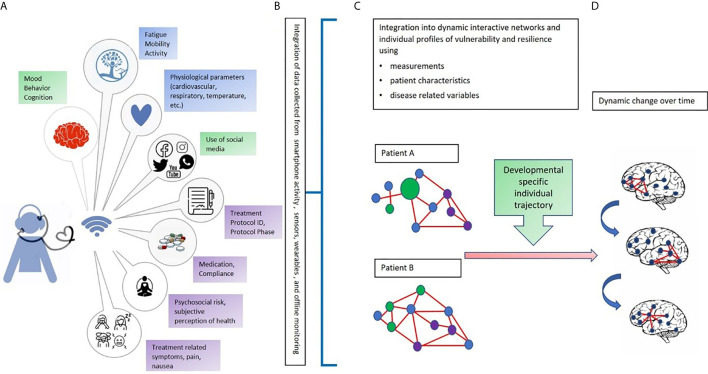
An integrated multi-dimensional model for the use of digital phenotyping as part of a digital “toolbox” to be used in adolescents with cancer. Our hypothetical model is defined over four phases: **(A)** The data collection phase: In green: Objective continuous non-invasive data collection related to mood, behavior, cognition, and use of social media (using a digital phenotyping app); in blue: Objective continuous non-invasive collection of biomarkers from sensors and wearables (heartrate, blood pressure, temperature); in purple: Periodic collection of subjective perceptions and impressions related to mental and physical wellbeing (e.g., diaries, self-reports, and questionnaires) and type of treatment, protocol, and protocol phase; **(B)** The data transformation phase, that is, the conversion of raw data into usable algorithms and outputs to feed data analysis and network construction; **(C)** Creation of individual networks based on data from different dimensions and domains to understand the strengths and needs of each patient. The circles represent signs, symptoms, and factors related to the domains of data collection (green, blue, and purple). The red lines represent the connections among signs, symptoms, and factors; **(D)** Development of dynamic networks to monitor change over time in network organization and connectivity aimed at identifying windows of vulnerability and resilience. Letters and colors refer to those shown in the figure.

Digital phenotypes, digital biomarkers, mobile asynchronous questionnaires, and self-reports provide data along various dimensions that, once collected, will reflect the complexity and delicacy of the behaviors of adolescent patients. Challenges also exist with this approach since to benefit from a digital analysis, very large amount of data reflecting the complexity of developmental and disease related factors that may alter individual behavior almost instantly must be factored in (**Figure 1**).

## Dynamic Mapping of Multidimensional Data

At present, evidence has accumulated that the engrained traditional approach linking just one or a few (often molecular or biological) mediators to an illness related phenotype, limits the understanding of the complex underlying pathologic conditions which has hampered progress in the development of efficient treatments.

To this end, traditional statistical approaches are not capable of capturing the particularity of each individual specific phenotype. At present, modern approaches coming from machine learning, especially those related to deep learning, provide innovative ways to tackle the complexities of multidimensional continuous data collection. The integration of data collected from the different instruments in a digital toolbox, ranging from sensor recorded physical functioning to digital phenotyping, need a more sophisticated approach, such as deep learning and network analysis. Recently, researchers have started to use deep neural networks in big-data rich areas such as online social media platforms or smartphone and mobile sensor-based data relating them to mental health (48–51).

Therefore, combining network science and dynamic system theory with a toolbox containing digital phenotyping and biomarkers permits the integration of data across diverse levels of analysis and capture the nature of their dynamic relationship over time, both for patients and treatment modalities (52–57) (**Figure 1**).

New conceptual thinking may result in increasingly explanatory and predictive models resulting in a more realistic image of the strengths and vulnerabilities of these young patients at high-risk for behavioral and emotional problems. Network analysis helps to determine if one domain or function is more important than others, if changes in one domain, factor, or symptom dynamically influence the function of others, and finally, what factor or domain is driving the network. In sum, network analysis will not only indicate which domains and factors mostly define individual vulnerability but will have important implications for clinical practice motivating personalized strategies in the prevention of mood, behavioral, and cognitive problems (46, 54–57).

## Inevitable Opportunities, Inevitable Challenges

Notwithstanding the promise to deliver a revolution in the health care of children and especially adolescents diagnosed with cancer, the incredible potential of the digital era comes with substantial questions regarding its implementation.

While artificial intelligence, deep learning, and network analyses sustain the analysis of complex data, their use requires the acquisition of innovative expertise and competence. The analysis of large multi-domain data bases needs a multidisciplinary collaborative effort which is currently lacking.

Today, more than in the past we are witnessing an increasing number of disruptive products such as advanced mRNA, gene and cell therapies that face unexpected legal and ethical challenges. Therefore, data must be managed in a thoughtful way. We must prepare for a profound change in the way data are gathered and integrated from multiple sources including digital tools. For digitally captured data, quality measures must be incorporated, and the sensitivity, specificity, accuracy, and precision of device parameters and measurements need to be tested.

Also, personal health-related data, especially from minors, should be considered in a meaningful way. If we want to engage young patients as data generators, a trusted ethical, legal, and regulatory ecosystem should be created with clear rules. Innovative, legal, organizational, and technical solutions are needed to share their sensitive, health-related personal data in an ethical and privacy-compliant environment.

Therefore, adopting this novel approach should be accompanied by adequate ethical and regulatory support to fully protect the patient and to overcome present and future challenges linked to confidentiality and accountability. While having the knowledge and the technical capacity to truly benefit from digital tools, widespread information, education, and training would be needed from both the clinician and the patient. Thus, we, adults, clinicians, and researchers need to step up to the challenge and avoid becoming the rate limiting step in embracing a new era (23, 24).

## Possible Impact

The impact of digital phenotyping and dynamic monitoring likely involves multiple domains of care as well as multiple stakeholders. Researchers will have to find new ways to enrich, share, and analyze data and develop strategies to support patient friendly access. At the same time research should direct efforts to the development of decision-making paradigms that better inform and sustain clinical interventions. For patients, the use of a digital toolbox represents an innovative way to study their needs and challenges while being treated for cancer. With the patient as active participants we will be better able to transform their needs into interventions that aim to support their wellbeing and prevent serious problems before they start. Fine-grained phenotypes representing the individual aspects of emerging problems may induce industry to develop new therapies and sustain faster progress from bench to bedside. Finally, an ecological day-to-day collection of data should stimulate regulators to identify new targets and determine when and how a target may become acceptable for regulatory decision making centered on the adolescent.

## With the Future of the Adolescent Patient in Mind

The care process surrounding the adolescent patients is an active, planned, coordinated, comprehensive, multidisciplinary, and multi-stakeholder process that should be flexible, developmentally appropriate considering the interaction and interdependency of medical, psychosocial, behavioral, and environmental factors. The use a digital toolbox combined with network analysis may define developmental trajectories of risk and resilience and reveal more fine-grained cognitive and behavioral phenotypes, that together with clinical factors, and biological markers, may explain the relationship between disease, age specific risk, and the efficacy of treatment. To achieve this, the combination of sufficiently diverse tools, by themselves not new, will feed complex datasets with broad as well as deep phenotypic representation of the patients’ needs.

## The Added Value of Entering the Digital Era

According to the suggested paradigm shift, each individual young patient will be involved in a process aimed to predict, prevent, and personalize their care, stressing the individual’s participation moving from a passive receiver of care to an active and conscious contributor to their wellbeing.

A “digital toolbox” which includes digital phenotyping will give us hope for the future (58) and will help to enrich the care of adolescent patients with cancer. Starting from each individual patient, and within known treatment modalities and protocols, we will be better able to recognize interrelated behaviors, risk factors, and treatment and disease related side effects and to isolate the domain(s) most central to possible adverse outcome. Combining pharmacovigilance and behavioral phenotyping with easily accessible technologies and the most innovative analyses will improve timely access to the detection of adverse medical and behavioral events. In turn, this will stimulate efforts to improve, develop, and implement innovative programs of personalized interventions adapted to the clinical needs and characteristics of individual patients. Finally, such a process, would fully respect the so-called P4 of Precision Medicine: predictive, preventive, personalized, and participatory, putting the adolescent patient at the very core of their present and future health.

## Author Contributions

JMCB and LP conceived the manuscript. JMCB wrote the first draft. LP, CB, CC, and FT critically revised the manuscript. All authors contributed to the article and approved the submitted version.

## Conflict of Interest

Author LP is a consultant for VeraSci.

The remaining authors declare that the research was conducted in the absence of any commercial or financial relationships that could be construed as a potential conflict of interest.
